# Blood pressure responses are dependent on call type and related to hypertension status in firefighters

**DOI:** 10.1080/08037051.2022.2161997

**Published:** 2023-12

**Authors:** Paige J. Rynne, Cassandra C. Derella, Carly McMorrow, Rachel L. Dickinson, Stephanie Donahue, Andrew A. Almeida, Megan Carty, Deborah L. Feairheller

**Affiliations:** a Department of Kinesiology, University of New Hampshire, Durham, NH, USA; b Department of Physiology, Augusta University, Augusta, GA, USA; c Misericordia University, Pittsburgh, PA, USA; d Philadelphia College of Osteopathic Medicine, Philadelphia, PA, USA; e Jefferson College of Population Health, Thomas Jefferson University, Philadelphia, PA, USA; f Department of Kinesiology, California State University San Marcos, San Marcos, CA, USA

**Keywords:** Ambulatory blood pressure, blood pressure surge, cardiovascular disease, firefighters, stress response, hypertension, alarm response

## Abstract

**Background:**

Impaired cardiovascular health is a concern for firefighters, with over 50% of line-of-duty deaths having cardiac causes. Many firefighters have hypertension and <25% have their blood pressure (BP) controlled. The alarm response could be an unidentified cardiac risk, but interestingly, the BP response to different calls and on-the-job activity is unknown.

**Purpose:**

We aimed to measure the physiological stress resulting from different call types (fire, medical) and job activity (riding apparatus, pre-alert alarms) through ambulatory BP (ABP) monitoring in a population of firefighters.

**Materials and methods:**

During 111 12-h work shifts firefighters wore an ABP monitor. BP was measured at 30-min intervals and manual measurements were prompted when the pager went off or whenever they felt stress.

**Results:**

Firefighters were hypertensive (124.3 ± 9.9/78.1 ± 6.7 mmHg), overweight (30.2 ± 4.6 kg/m^2^), middle-aged (40.5 ± 12.6 years) and experienced (17.3 ± 11.7 years). We calculated an average 11% increase in systolic and 10.5% increase in diastolic BP with alarm. Systolic BP (141.9 ± 13.2 mmHg) and diastolic BP (84.9 ± 11.1 mmHg) and the BP surges were higher while firefighters were responding to medical calls compared to fire calls. Between BP groups we found that medical call systolic BP (*p* = .001, *d* = 1.2), diastolic BP (*p* = .017, *d* = 0.87), and fire call systolic BP (*p* = .03, *d* = 0.51) levels were higher in the hypertensive firefighters.

**Conclusion:**

This is the first report of BP surge responses to alarms and to occupational activities in firefighters, and medical calls elicited the largest overall responses.

## Introduction

Cardiovascular disease (CVD) continues to be a source of extreme health and economic burden. Cardiac-related incidents are critical issues in the military, in veterans, and in firefighters. It is known that CVD is the leading cause of mortality in the world with many modifiable risk factors, including elevated blood pressure (BP), high total cholesterol, smoking, diabetes, obesity, and physical inactivity [1]. Based on the new guidelines for hypertension diagnosis, a larger percentage of the population is identified as hypertensive [2]. Thus, the clinical use of ambulatory BP (ABP) monitoring is becoming a valuable tool for accurate diagnosis, management, and treatment of hypertension [2]. It is believed that ABP measurements may better predict target organ damage from hypertension compared to brachial BP measured in the office or clinic [3,4]. To better understand the aetiology of BP, studies are needed in a variety of occupational groups, including tactical populations, that examine ABP responses to stimuli [5,6].

Throughout the day, certain activities can trigger a stress response which temporarily elevates BP. This response is known as a BP surge. Additionally, it is known that BP fluctuates, and a higher BP variability has prognostic value in predicting risk and future cardiac events [7]. The lack of BP awareness and correspondingly high levels of hypertension remain public health issues. Current military studies have reported that CVD risk is on the rise in younger active and reserve service members, and veterans still have worse health profiles than their active-duty counterparts [8]. Also, CVD and impaired cardiovascular health are noticeably more prevalent in firefighters than in civilians. In firefighters, over 50% of line-of-duty deaths are cardiac-related [9]. Many firefighters have hypertension, but <25% have their BP under control [10]. A recent study estimated that 39% of firefighters have elevated levels of BP, 45% may fall in the Stage I and Stage II hypertensive categories, and 50% of firefighters do not know their BP [11]. We have previously reported that firefighters have exaggerated BP responses to a workload while wearing protective gear [12], but no study has examined BP responses to the occupational activity.

The increased CVD risk and higher levels of hypertension seen in firefighters may be a combination of physical and emotional stress due to the nature of the job. In prior studies, it has been shown that there is a direct relationship between work stress and BP [13,14]. Firefighters and emergency service personnel have some of the most stressful jobs on a day-to-day basis due to their occupational demands [15,16]. Firefighting is an occupation characterised by stress, exposure to harmful agents, hazardous health risks, pressure-filled situations, and immediate response to alarms. It is known that an alarm sound causes an instant sympathetic nervous system response where an immediate BP and heart rate spike occurs, and it is thought that this could be a contributor to the line-of-duty death incidence in firefighters [16].

Anecdotal reports from firefighters and clinicians include comments like ‘I know my BP spikes when the pager goes off’, and ‘I can feel my heart pounding’. Therefore, we aimed to evaluate and quantify the physiological stress resulting from different types of calls (fire, medical) and job activity (riding apparatus, pre-alert alarms in the firehouse) through ABP monitoring in a population of firefighters. Pre-alert systems are designed to alert firefighters of an incoming emergency dispatch call with the thought that a lighter or softer sound would be less ‘stressful’, and these pre-alert alarms are sounded in some firehouses. We hypothesised that fire calls would result in the largest BP surge in firefighters compared to other types of calls and work conditions. We also hypothesised that hypertensive firefighters would have exaggerated cardiovascular responses.

## Materials and methods

One hundred thirty-three firefighters were recruited from fire stations in the Philadelphia PA and Durham NH areas. From this, eight did not meet the BP measurement adherence threshold (70% during the 12-h timeframe), four never had a 911 emergency call occur, and 10 chose not to wear an ABP monitor for the full 12 h. Therefore, we present data on 111 (100 M, 11 F) firefighters. This is a similar gender ratio (9.9% female) to the overall firefighter population which has been reported by the National Firefighter Protection Agency (10% female) [17]. Any firefighters with history of diabetes or CVD were excluded. During their first visit, each firefighter received a full explanation of the study protocol, completed a health history form, and provided written informed consent. The protocol was approved by the Ursinus College and the University of New Hampshire Institutional Review Boards.

Height and weight were measured using a stadiometer and digital floor scale (BC-533; Tanita Corporation, Arlington Heights, IL, USA) without shoes. Body mass index (BMI) was calculated and recorded. Clinical BP measurements were collected in accordance with guidelines following 5 min of rest. Multiple measurements were made several minutes apart over at least 3 days, and the average of all readings is reported as clinical BP.

ABP monitoring was completed as previously described [18]. Non-invasive portable ABP monitors (SpaceLabs, Model 90127, Redmond, WA, USA) were worn by each firefighter for a 12-h period. BP measures were obtained at 30-min intervals during the daytime period (6:00 a.m.–10:00 p.m.), and 60-min intervals at night (10:00 p.m.–6:00 a.m.) if worn during that timeframe. Firefighters were all instructed to wear the monitor for at least 12 h. Some firefighters chose to wear the monitor for longer than 12 h so that they could collect data for their own records, but we only analysed the data from their 12-h work shift. When an emergency call came in and their pager went off, firefighters were instructed to push the monitor’s button to initiate an automatic BP reading. They were also asked to force BP readings when they were riding the apparatus, when the pre-alert alarm would sound in the firehouse, or at any other time that they felt ‘stress’ at work. These extra readings that were collected enabled our detailed analysis and were in addition to the normal 12-h ambulatory monitoring. We report here the data collected during fire calls, medical calls, riding the apparatus, and pre-alert alarm readings. Additionally, all firefighters enrolled in the study were asked to keep a log while wearing the ABP monitor. They were given a data sheet where they self-reported information on the time of day for each reading, what they were doing, how they were feeling, and what type of emergency call or work activity they were performing. The BP measurements collected over the 12-h monitoring period were averaged and the mean value is reported as ABP. The BP surge measurements collected upon alarm or under stress were analysed in comparison to the immediate BP reading taken at the time of the event. For comparison, the BP measurements collected for each condition were averaged by group and are presented as apparatus BP, medical BP, fire call BP, and pre-alert BP.

### Statistical analysis

The results are expressed as mean values ± standard deviation, and significance was set at *p* < .05. For the report of ABP measurements, all ABP readings were averaged, for the 12-h period and for each condition, to give a single systolic and diastolic BP measure. Data from firefighters who collected <70% of the potential ABP readings during their 12-h work shift were excluded. The number of potential ABP readings was determined based on the 12-h timeframe when a firefighter wore the monitor, excluding any additional readings that they may have been collected on the job. The values of systolic and diastolic BP readings were analysed separately. The distribution of all variables was examined using Shapiro–Wilk test of normality. Differences between hypertensive and normotensive firefighters were evaluated using the independent sample *t*-test. Differences in BP readings by occupational condition were assessed by one-way analysis of variance (ANOVA). The comparison of BP values within the group of firefighters between different call types or occupational activity were analysed with paired *t-t*est. Data was covaried for gender, age, and prior BP medication usage. The effect size for analysis was examined using Cohen’s *d*. Correlations (Pearson, two-tailed) were calculated to examine relationships between BP measures. SPSS 28.0.1 (SPSS Inc., Chicago, IL, USA) was used for all analyses.

## Results

Clinical characteristics are presented in Table 1. One hundred and eleven data points were included in the analysis which reports on 2930 total BP readings, with an average of 26.4 BP readings per 12-h shift. During the 111 shifts, firefighters logged 186 total calls that were captured by ABP monitoring. Data included is from 100 (90.1%) male firefighters and 11 (9.9%) female firefighters. Firefighters reported an average of 17.3 years of service as a municipal firefighter, with 56 (50.5%) reporting more than 15 years of service. In all, 74 (66.7%) of the firefighters in our study were hypertensive according to clinical seated BP guidelines. Furthermore, 12 firefighters had stage 2 hypertension, with a seated resting BP of ≥140 mmHg systolic or ≥90 mmHg diastolic. Firefighters were informed of the clinical BP measurements that were collected. If they were hypertensive, it was suggested that they follow-up with their clinician. Anecdotal reports from some of the participants were that they started to monitor their own BP levels while on shift, so the study raised awareness within the crews of the increased risk of hypertension. Between the hypertension groups, height (*p* = .02, *d* = 0.49), weight (*p* = .00, *d* = 1.0), and BMI (*p* = .00, *d* = 0.76) were higher in the hypertensive firefighters.

Blood pressure measurements are presented in Table 2. Based on clinical guidelines, the entire group on average is classified as hypertensive which matches previous reports of hypertension in firefighters [2,10]. Considering all of the BP measurements, we found an average 11% increase in systolic and 10.5% increase in diastolic BP with alarm, which corresponds to the calculated systolic BP surge of 17.1 mmHg and diastolic BP surge of 10.3 mmHg with the immediate measurement when pager sounds. Hypertensive firefighters seemed to have higher BP surge measurements than normotensive firefighters, but this was not significant. We found that clinical systolic (*p* = 0.00, *d* = 2.2) and diastolic BP (*p* = .00, *d* = 1.1), average ambulatory systolic (*p* = .00, *d* = 0.9) and diastolic BP (*p* = .002, *d* = 0.62), and ambulatory mean arterial pressure (*p* = .001, *d* = 0.67) were higher in the hypertensive firefighters.

**Figure 1.**
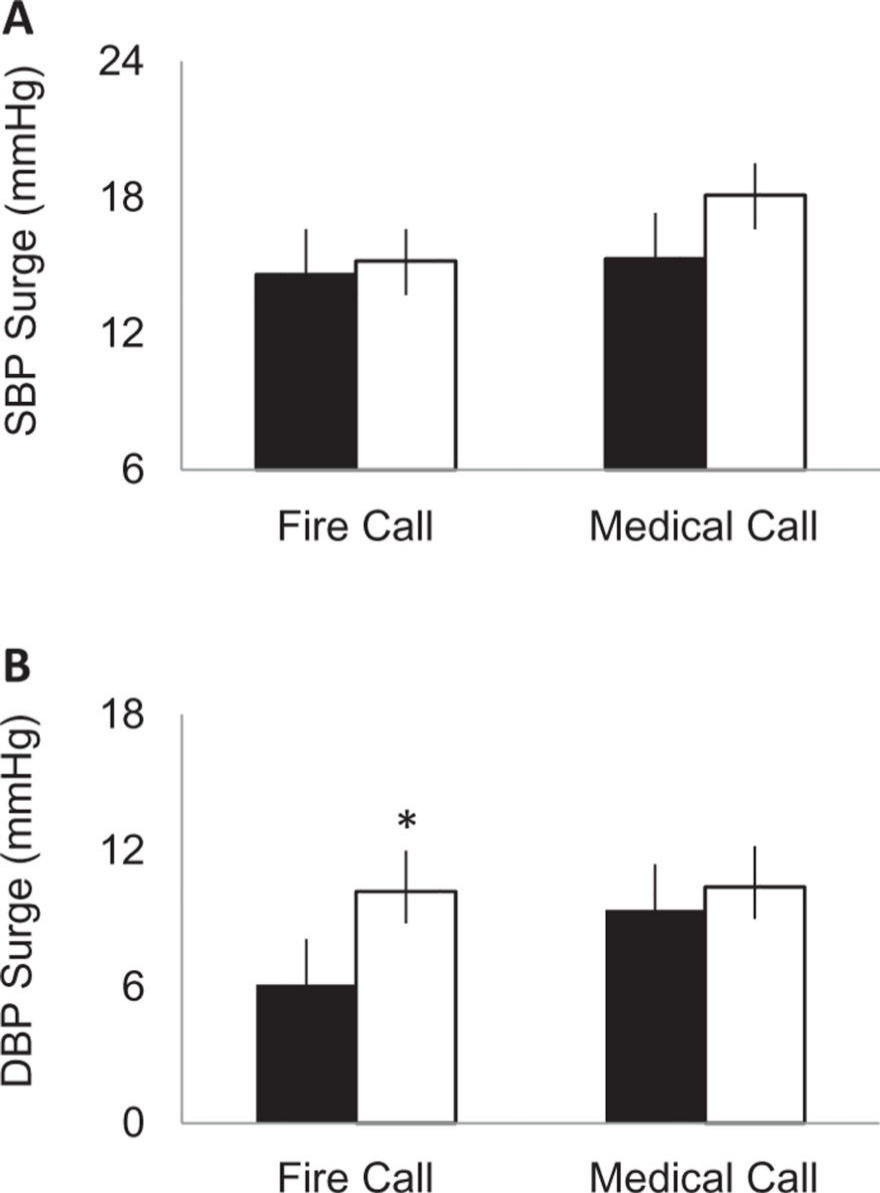
Blood pressure surge measured by call-type. Data shows (A) systolic blood pressure (SBP) surge and (B) diastolic blood pressure (DBP) surge values in normotensive firefighters (solid bars) compared to hypertensive firefighters (open bars). BP surge values capture the BP immediately occurring when the pager alarm sounds. *p < .05 between groups.

Figure 1 shows the comparison between hypertensive and normotensive firefighter responses to fire and medical calls. Overall, medical calls appeared to elicit higher BP responses than fire calls. Between group analysis showed that systolic and diastolic BP surges were higher in hypertensive firefighters for both fire and medical calls, with only the diastolic BP surge significantly higher between groups for fire calls (*p* = .014, *d* = 0.51).

BP measurements collected by the condition are presented in Table 3. The average blood pressure level measured was dependent on call or activity type. Based on the unpredictable nature of the job, firefighters were able to collect different numbers of BP measurements. Table 3 reports the average values from all readings collected while riding apparatus (10 in normotensive and 23 measures collected in hypertensive firefighters), on a medical call (11 in normotensive and 33 measures collected in hypertensive firefighters), on a fire call (32 in normotensive and 54 measures collected in hypertensive firefighters), and with the pre-alert (three in normotensive and 14 measures collected in hypertensive firefighters). Within the entire group, we found that BP measured in response to medical calls was the highest and BP measured while firefighters rode apparatus was the lowest. In the group, systolic BP on a medical call was higher than systolic BP (*p* = .001, *d* = 0.88) on the apparatus, diastolic BP on a medical call was higher than diastolic BP (*p* = .02, *d* = 0.54) taken on the apparatus, and systolic BP on a fire call was higher than the systolic BP (*p* = .04, *d* = 0.38) measured on the apparatus. As shown in Table 3, between BP groups we found that medical call systolic BP (*p* = .001, *d* = 1.2), diastolic BP (*p* = .017, *d* = 0.87), and fire call systolic BP (*p* = .03, *d* = 0.51) levels were higher in the hypertensive firefighters compared to normotensive firefighters.

**Figure 2.**
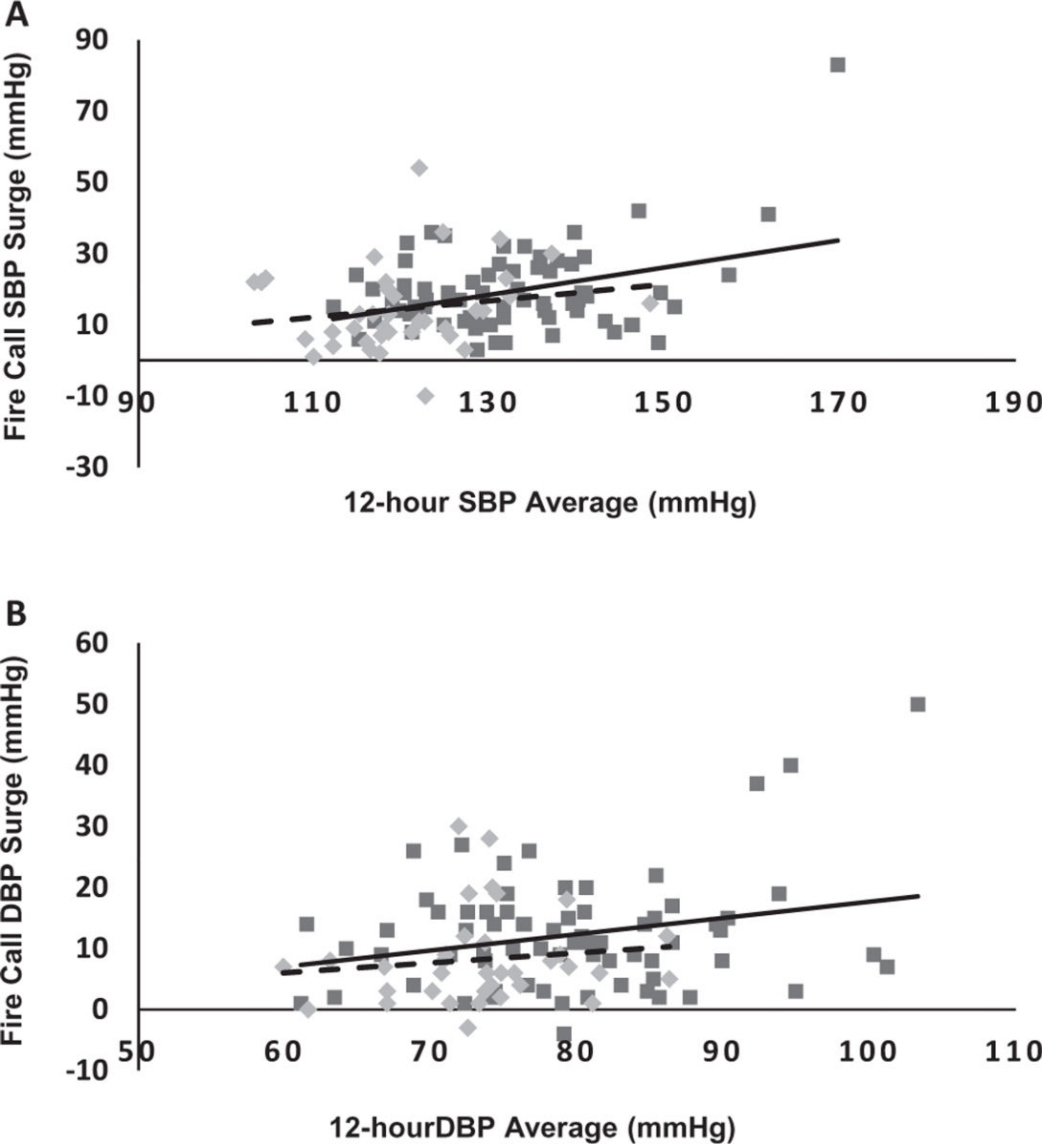
Comparison of the relationship between (A) systolic blood pressure (SBP) and (B) diastolic blood pressure (DBP) surge levels and 12-h ambulatory blood pressure averages. Data shows the linear trend in hypertensive firefighters (Solid line, dark grey squares) and normotensive firefighters (dashed line, light grey diamonds). BP surge values capture the BP immediately occurring when the pager alarm sounds. *Both significant at *p* = .01.

Figure 2 shows the correlation scatterplot figures for both normotensive and hypertensive firefighters. We found a stronger relationship in hypertensive firefighters (*r* = .824, *p* = .00) than in normotensive firefighters (*r* = .29, *p* = .11) between the systolic BP surge in response to fire calls with the overall 12-h systolic ABP average. We also found a stronger relationship between 12-h diastolic ABP values and diastolic BP surge in hypertensive firefighters (*r* = .529, *p* = .00) than in normotensive firefighters (*r* = .19, *p* = .29).

## Discussion

This is the first report of measured BP surge values to 911 alarms in a group of municipal firefighters. The purpose of this study was to quantify the physiological stress resulting from different types of calls (fire and medical) and job activity (riding apparatus and pre-alert alarms in the firehouse) through ABP monitoring in a population of firefighters. The main findings of this study are that the average BP level measured is dependent on call or activity type. We hypothesised that fire calls would result in the largest BP surge in firefighters compared to other types of calls and work conditions, but this was not true. We found that medical calls yielded the highest overall BP readings, and BP measured while firefighters rode apparatus was the lowest. We also hypothesised that hypertensive firefighters would have exaggerated responses, and this was confirmed. In our study, the hypertensive firefighters had higher BP levels and appeared to have larger BP surges with alarm. Furthermore, it seems that high BP in any firefighter has a direct relationship with the average ABP level.

Recently we summarised the literature on BP responses in firefighters and found that there is a gap in the literature examining BP response to alarm [19]. The tactical operations and hazardous nature of firefighting influence the stress responses and therefore affect BP. Considering that hypertension in firefighters often remains undiagnosed or undocumented, studies like this are valuable. Typically, the work of firefighting, regardless of volunteer or career, involves long periods of inactivity followed by unpredictable and sometimes physically demanding work, accompanied by life-threatening activity. Evidence also suggests that such strenuous stimuli could result in cardiac-related events [20]. It is known that alarm response, which is like the body’s fight-or-flight response, is related to the risk of cardiac events around 10 times higher than the work of firefighting [21]. Medical calls elicited the largest response from our firefighters, and future studies should examine the psychosocial aspect of firefighting. As this is the first report of BP surges, it needs to be examined why medical calls had higher overall BP readings.

Recent guidelines continue to advocate the use of ABP monitoring for hypertension diagnosis [5,6]. BP measurements collected in the office provide a single reading at one-time point of patient’s BP, so this one measurement may be less predictive of CVD risk. Ambulatory monitoring allows assessment of daytime BP, night-time BP, morning BP surge levels, and BP levels during daily or occupational activities. ABP may also be a clinical way to assess psychological stress [15]. Also, it has been suggested that the risk of sudden cardiac death in firefighters may increase 5–7 times during the alarm response compared to non-emergency time [21,22]. Prior research has found that higher anxiety and stress exist in firefighters within the first minute of alarm exposure which may continue throughout the extreme work of firefighting [23]. The alarm sound activates the sympathetic nervous system which is also related to an increase in sudden cardiac events during alarm responses [24]. In our firefighters, BP surge with medical calls was higher for both systolic and diastolic BP levels. Prior studies have found that firefighters with post-traumatic stress disorder (PTSD) experience an increased physiological stress response when exposed to a traumatic stimulus [25]. We speculate that the exaggerated BP surge in response to medical calls is likely due to the anticipation of traumatic events based on past experiences, and the nature of medical calls.

We saw that hypertensive firefighters seemed to experience higher BP surges. Hypertension remains the leading risk factor for CVD with exaggerated morning BP surge in hypertensives having a strong relationship with CVD. It can be argued that BP alarm response to 911 pager tones in firefighters is similar to an exaggerated BP surge, much like a morning BP surge [26]. Prospective research has demonstrated that morning BP surge is a risk for cardiac events, but it has yet to be determined whether the BP surge that emergency service personnel experience is related to CVD [27]. Furthermore, BP surge differences relate to BP variability and multiple definitions exist for morning BP surge and BP variability. Prior research has found that higher morning BP surge is related to left ventricular mass and ejection fraction ratios in older patients, while in firefighters it has been reported that body composition is the only consistently significant predictor of left ventricular mass indexes [28,29]. We are the first to report that firefighters with hypertension may have heightened responses to alarm. This should be investigated further. The relationship between BP variability, BP surge with alarm, and cardiac risk needs to be evaluated in future studies.

A limitation of this research is that the study groups were in the Philadelphia PA and Durham NH regions, both being suburban areas. Also, all firefighters who participated were municipal firefighters. Therefore, the data presented may not be representative of firefighters in rural or urban areas or of the wildland urban-interface firefighters. The gender of participants in this study was unbalanced, with only 11 of the 111 firefighters being female. However, this gender ratio (9.9% female) is aligned with the gender ratio of the American firefighting population (10% female) [17]. Firefighter attributes, such as alarm fatigue, years of experience, hydration status, mental state, sleep status, and fitness level, were not taken into consideration since this was the first data collection of this type. Previous studies have found that emergency alarms during the night elicit a larger HR surge and cortisol response in comparison to alarms during the day [30]. In future studies, it would be beneficial to evaluate how these additional variables affect the intensity of the cardiovascular response to different call types and job activities in firefighters.

In conclusion, the immediate increases in BP seen with alarm are a cause for concern given the impaired cardiovascular health of the average firefighter. Specifically, firefighters who are clinically hypertensive should be aware of their own BP levels, since it seems that their BP levels during calls are exaggerated. Further research is needed to elucidate potential physiological relationships with cardiac-related biomarkers and the BP response in firefighters to different call types and activities. Additionally, it would be beneficial to evaluate the effect of psychological factors, such as prior exposures to traumatic events, on the cardiovascular response. Ascertaining how these factors impact the magnitude of the stress response is essential, as we hope this information will help identify ways in which the BP surge in firefighters, and the resulting risk of adverse cardiac events, can be reduced.

## Figures and Tables

**Table 1.** Firefighter characteristics.

	Entire group	Normotensive FF	Hypertensive FF
Age (years)	40.5 (12.6)	37.1 (13.7)	42.1 (11.9)
# years as FF	17.3 (11.7)	16.1 (11.4)	17.9 (11.9)
Height (cm)	175.0 (7.4)	172.6 (7.5)	176.2 (7.2)*
Weight (kg)	91.3 (14.6)	82.1 (13.4)	95.8 (13.1)*
BMI (kg·m^−2^)	30.2 (4.6)	27.9 (4.1)	31.2 (4.3)*
# BP measures	26.4 (9.5)	25.8 (9.2)	26.7 (9.6)

FF: firefighter; BMI: body mass index;

#BP measures: # blood pressure measurements collected.

Data are presented as mean (*SD*) for 111 total firefighters; 37 normotensive, 74 hypertensive.

* Significant at *p* < .05 between groups.

**Table 2.** Blood pressure measurements.

	Entire group	Normotensive FF	Hypertensive FF
Clinical SBP, mmHg	124.3 (9.9)	114.1 (4.9)	129.4 (7.6)*
Clinical DBP, mmHg	78.1 (6.7)	73.6 (5.3)	80.4 (6.1)*
Ambulatory SBP, mmHg	127.8 (12.1)	120.5 (9.4)	131.4 (11.7)*
Ambulatory DBP, mmHg	77.8 (8.5)	74.4 (7.1)	79.5 (8.7)*
Ambulatory MAP, mmHg	93.5 (9.2)	89.5 (7.5)	95.5 (9.5)*
SBP surge, mmHg	17.1 (12.1)	14.8 (12.1)	18.2 (12.1)
DBP surge, mmHg	10.3 (9.3)	8.4 (7.4)	11.2 (9.9)

FF: firefighter; SBP: systolic; DBP: diastolic blood pressure; MAP: mean arterial pressure.

Data are presented as mean (*SD*) for 111 total firefighters; 37 normotensive, 74 hypertensive.

* Significant at *p* < .05 between groups.

**Table 3.** Blood pressure by occupational condition.

	Entire group	Normotensive FF	Hypertensive FF
Apparatus SBP, mmHg	135.9 (12.2)	130.8 (13.9)	138.1 (10.9)
Apparatus DBP, mmHg	81.8 (10.2)	78.0 (8.5)	83.4 (10.6)
Medical call SBP, mmHg	141.9 (13.2)	131.0 (9.9)	145.6 (12.2)*
Medical call DBP, mmHg	84.9 (11.1)	78.1 (9.1)	87.2 (10.9)*
Fire call SBP, mmHg	138.4 (18.7)	132.6 (18.0)	141.8 (18.3)*
Fire call DBP, mmHg	83.7 (12.2)	80.5 (10.6)	85.6 (12.7)
Pre-alert SBP, mmHg	137.4 (10.5)	129.0 (6.9)	139.2 (10.4)
Pre-alert DBP, mmHg	83.3 (8.2)	85.6 (9.1)	82.8 (8.2)

FF: firefighter; SBP: systolic blood pressure; DBP: diastolic blood pressure. Data are presented as mean (*SD*) for 111 total firefighters; 37 normotensive, 74 hypertensive.

* Significant at *p* < .05 between groups.

## Data Availability

The datasets generated and analysed during the current study are available from the corresponding author on reasonable request.

## PLAIN LANGUAGE SUMMARY

- Cardiovascular disease and impaired cardiovascular health are substantially more prevalent in firefighters, with over 50% of line-of-duty deaths being cardiac related.
- Many firefighters are diagnosed with high blood pressure (hypertension), which is known to increase the risk of heart attacks, strokes, heart disease, and other serious health complications.
- Upon stress, our body enacts the ‘fight or flight’ response where sympathetic nervous system activity triggers an immediate increase in heart rate and blood pressure. This response can be dangerous when surges reach extreme levels due to underlying impaired cardiovascular function. It is known that alarm sounds trigger a stress response.
- Firefighters respond to different alarms while on the job, each indicating different call types, such as a house fire or a medical emergency. Due to the prevalence of impaired cardiovascular health in firefighters, the physical stress resulting from these alerts is cause for concern.
- The blood pressure surge response to different call types and job activities in healthy and hypertensive firefighters had not been measured before this study.
- Through the ambulatory blood pressure monitoring of 111 on-duty firefighters, this study discovered that medical calls caused the greatest blood pressure and heart rate surge.
- Also, firefighters with hypertension experienced a greater blood pressure surge in response to alarms than their non-hypertensive co-workers.

## References

[1] Benjamin EJ, Blaha MJ, Chiuve SE, Heart disease and stroke statistics-2017 update: a report from the American Heart Association. Circulation. 2017; 135(10):e146–e603. 28122885 10.1161/CIR.0000000000000485 PMC5408160

[2] Whelton PK, Carey RM, Aronow WS, 2017 ACC/AHA/AAPA/ABC/ACPM/AGS/APhA/ASH/ASPC/NMA/PCNA guideline for the prevention, detection, evaluation, and management of high blood pressure in adults: executive summary: a report of the American College of Cardiology/American Heart Association Task Force on clinical practice guidelines. J Am Coll Cardiol. 2018;71(19): 2199–2269. 29146535 10.1016/j.jacc.2017.11.006

[3] Kikuya M, Ohkubo T, Asayama K, Ambulatory blood pressure and 10-year risk of cardiovascular and non-cardiovascular mortality: the Ohasama study. Hypertension. 2005;45(2):240–245. 15596571 10.1161/01.HYP.0000152079.04553.2c

[4] Verdecchia P. Prognostic value of ambulatory blood pressure: current evidence and clinical implications. Hypertension. 2000;35(3):844–851. 10720605 10.1161/01.hyp.35.3.844

[5] Dolan E, O’Brien E. How should ambulatory blood pressure measurement be used in general practice? J Clin Hypertens. 2017;19(3):218–220. 10.1111/jch.12952 PMC8031091 27991714

[6] O’Brien E, Parati G, Stergiou G. Ambulatory blood pressure measurement: what is the international consensus? Hypertension. 2013;62(6):988–994. 24060895 10.1161/HYPERTENSIONAHA.113.02148

[7] Blacher J, Safar ME, Ly C, Blood pressure variability: cardiovascular risk integrator or independent risk factor? J Hum Hypertens. 2015;29(2):122–126. 24990422 10.1038/jhh.2014.44

[8] Hoerster KD, Lehavot K, Simpson T, Health and health behavior differences US military, veteran, and civilian men. Am J Prev Med. 2012;43(5): 483–489. 23079170 10.1016/j.amepre.2012.07.029

[9] Geibe JR, Holder J, Peeples L, Predictors of on-duty coronary events in male firefighters in the United States. Am J Cardiol. 2008;101(5):585–589. 18308003 10.1016/j.amjcard.2007.10.017

[10] Soteriades ES, Kales SN, Liarokapis D, Prospective surveillance of hypertension in firefighters. J Clin Hypertens. 2003;5(5):315–320. 10.1111/j.1524-6175.2003.02058.x PMC8101863 14564131

[11] Risavi BL, Staszko J. Prevalence of risk factors for coronary artery disease in Pennsylvania (USA) firefighters. Prehosp Disaster Med. 2016;31(1):102–107. 26680533 10.1017/S1049023X15005415

[12] Feairheller DL. Blood pressure and heart rate responses in volunteer firefighters while wearing personal protective equipment. Blood Press Monit. 2015;20(4):194–198. 25856421 10.1097/MBP.0000000000000120

[13] Andel SA, Pindek S, Spector PE. Being called to safety: occupational callings and safety climate in the emergency medical services. J Occup Environ Med. 2016;58(12):1245–1249. 27930486 10.1097/JOM.0000000000000899

[14] Vrijkotte TG, van Doornen LJ, de Geus EJ. Effects of work stress on ambulatory blood pressure, heart rate, and heart rate variability. Hypertension. 2000; 35(4):880–886. 10775555 10.1161/01.hyp.35.4.880

[15] Kaikkonen P, Lindholm H, Lusa S. Physiological load and psychological stress during a 24-hour work shift among Finnish firefighters. J Occup Environ Med. 2017;59(1):41–46. 28045796 10.1097/JOM.0000000000000912

[16] Rosenthal T, Alter A. Occupational stress and hypertension. J Am Soc Hypertens. 2012;6(1):2–22. 22024667 10.1016/j.jash.2011.09.002

[17] Haynes HJG, Stein P. US Fire Department Profile-2015. NFPA Research; 2017. p. 1–47.

[18] Diaz KM, Veerabhadrappa P, Kashem MA, Relationship of visit-to-visit and ambulatory blood pressure variability to vascular function in African Americans. Hypertens Res. 2012;35(1):55–61. 21814215 10.1038/hr.2011.135 PMC3629695

[19] McMorrow C, Feairheller DL. Blood pressure responses in firefighters reviewed. Curr Hypertens Rev. 2022;18(2):145–152. 34979891 10.2174/1573402118666220103094201

[20] Mittleman MA, Maclure M, Tofler GH, Triggering of acute myocardial infarction by heavy physical exerction. Protection against triggering by regular exertion. N Engl J Med. 1993;329(23): 1677–1683. 8232456 10.1056/NEJM199312023292301

[21] Kales SN, Soteriades ES, Christoudias SG, Firefighters and on-duty deaths from coronary heart disease: a case control study. Environ Health. 2003; 2(1):14. 14613487 10.1186/1476-069X-2-14 PMC293431

[22] Kales SN, Soteriades ES, Christophi CA, Emergency duties and deaths from heart disease among firefighters in the United States. N Engl J Med. 2007;356(12):1207–1215. 17377158 10.1056/NEJMoa060357

[23] Bugajska J, Zuzewicz K, Szmauz-Dybko M, Cardiovascular stress, energy expenditure and subjective perceived ratings of fire fighters during typical fire suppression and rescue tasks. Int J Occup Saf Ergon. 2007;13(3):323–331. 17888240 10.1080/10803548.2007.11076730

[24] Smith DL, DeBlois JP, Kales SN, Cardiovascular strain of firefighting and the risk of sudden cardiac events. Exerc Sport Sci Rev. 2016; 44(3):90–97. 27111479 10.1249/JES.0000000000000081

[25] Kales SN, Tsismenakis AJ, Zhang C, Blood pressure in firefighters, police officers, and other emergency responders. Am J Hypertens. 2009;22(1):11–20. 18927545 10.1038/ajh.2008.296

[26] Bombelli M, Fodri D, Toso E, Relationship among morning blood pressure surge, 24-hour blood pressure variability, and cardiovascular outcomes in a white population. Hypertension. 2014; 64(5):943–950. 25156170 10.1161/HYPERTENSIONAHA.114.03675

[27] Kario K. Morning surge in blood pressure and cardiovascular risk. Evidence and prespectives. Hypertension. 2010;56(5):765–773. 20937968 10.1161/HYPERTENSIONAHA.110.157149

[28] Kuwajima I, Mitani K, Miyao M, Cardiac implications of the morning surge in blood pressure in elderly hypertensive patients: relation to arising time. Am J Hypertens. 1995;8(1):29–33. 7734093 10.1016/0895-7061(94)00154-4

[29] Korre MS, Guilherme LGP, Farioli A, Effect of body mass index on left ventricular mass in career male firefighters. Am J Cardiol. 2016;118(11):1769–1773. 27687051 10.1016/j.amjcard.2016.08.058 PMC5312771

[30] Hall SJ, Aisbett B, Tait JL, The acute physiological stress response to an emergency alarm and mobilization during the day and at night. Noise Health. 2016;18(82):150–156. 27157688 10.4103/1463-1741.181998 PMC4918669

